# Sound effect of transmitting specific emotions in music performance based on the acoustic properties of conjugated materials

**DOI:** 10.3389/fchem.2023.1286318

**Published:** 2023-10-18

**Authors:** Yangjingyi Li, Fenghsu Lee

**Affiliations:** Faculty of Creative Arts, University of Malaya, Kuala Lumpur, Malaysia

**Keywords:** conjugated material, musical performance, emotional transmission, acoustic characteristic, sound effects

## Abstract

In music performances, instruments made of traditional materials often cannot accurately express specific emotions through means such as tone, pitch, and timbre, and it is difficult to adjust the sound effects according to specific emotions. This article studies the impact of conjugated materials on the timbre, frequency response, etc. of musical instruments, hoping to achieve sound effects that better convey specific emotions. This article analyzes the acoustic properties of conjugated materials and applies them to musical performances. It is found that conjugated materials can provide a wider selection of tones, pitches and timbres, performers can choose appropriate tone, pitch, and timbre from conjugated materials to express emotions according to specific emotional needs, or adjust and transform the tone, pitch, and timbre for specific emotions, thereby arousing emotional resonance from the audience, so that specific emotions can be conveyed to the audience more accurately and powerfully. At the same time, this research also found that compared with an environment with poor sound insulation, the average sound absorption coefficient of metal materials in a room with good sound insulation increased by 6.5%, and the average sound absorption coefficient of conjugate materials increased by 25.4%, which shows that the increase in the average sound absorption coefficient of conjugate materials is much greater than that of metallic materials. The research shows that studying the sound effects of musical performances based on the acoustic properties of conjugated materials will help explore and develop more immersive and emotionally rich musical experiences.

## 1 Introduction

Music is an important component of human culture, which can evoke emotional resonance and convey emotional information. At the same time, the sound effects in music performance also have a significant impact on conveying specific emotions. With the development of the music industry and the advancement of technology, people’s pursuit of sound effects has become increasingly high. Therefore, how to convey specific emotions through sound effects has become an important issue in music performance. Conjugated materials have unique acoustic properties, which can improve sound effects by adjusting the resonance frequency and sound intensity. In music, the application of conjugated materials in instrument manufacturing can achieve precise adjustment and control of sound. Through this method, each note in the music can convey specific emotions and improve the performance of the music.

By cleverly applying sound effects, the expressive power of music can be enhanced, allowing the audience to feel the emotions conveyed by the music more deeply. In order to understand the impact of music on people’s psychological activities, Hu Yanfang analyzed the similarities and differences between Chinese and Western music, as well as the inducing effects of music with different attributes on people’s emotions, providing theoretical support for the topic of music changing emotions (Hu, 2018). Music is an art and a common language of humanity. In order to better evoke emotional resonance in music, Lan Tianming proposed to grasp the emotions in singing and clarify the creative intention of the creators. Based on the actual situation of popular music singing, she explained how to more effectively achieve emotional control, in order to further highlight the value of popular music and let the songs bloom their charm (Lan, 2019). In order to achieve different integration effects by integrating various emotions into song singing, Lu Yidan analyzed the relationship between emotions and song singing, and determined the important role of emotions in song singing. Finally, she further explored ways to control emotions and improve the quality of singing (Lu, 2019). The importance of sound effects in conveying emotions in music performance is reflected in enhancing emotional resonance, enhancing expressive and infectious power, creating atmosphere and scenes, emphasizing and highlighting key emotions, and so on.

Conjugated materials, due to their unique acoustic properties and controllability, have greater potential in conveying specific emotional sound effects in music performance. Polyurethane sound absorption material is a commonly used porous foam sound absorption material, and its sound absorption performance is closely related to the microstructure. In order to apply it to the sound control of musical instruments, Li Yu analyzed the sound absorption coefficient of foam material at low, medium and high frequencies using the equivalent fluid model. The results showed that porous foam with rigid frame and high porosity has better sound absorption performance (Yu and Wen, 2022). Microporous sound-absorbing materials have been widely used in the field of music noise reduction, but the absorption performance of single-layer microporous plates is not enough to compete with porous materials. In order to improve the sound absorption performance of single-layer microporous plates, Gai X L introduced a composite structure of membrane cells and mass blocks. The sound absorption performance of microporous plates with composite structures of membrane cells and mass blocks was studied through impedance tube experiments. The results indicated that after the addition of membrane cells and mass blocks, the sound resistance of the structure increased, and the membrane cells and mass blocks could change the sound absorption characteristics of the microporous plate by introducing additional absorption peaks and valleys (GaiXingLi et al., 2018). Gong J synthesized diatomaceous earth polypropylene composite materials with excellent sound absorption performance using diatomaceous earth, polypropylene, foaming agent, and porous agent as precursor materials. He evaluated the sound absorption performance of the composite materials using a transfer function impedance tube sound absorption testing system. The results indicated that it has excellent compressibility, with a sound absorption coefficient of 0.85 (Gong, 2018). The acoustic properties of conjugated materials provide musicians and performers with a broader expressive space, enabling them to better present the emotions and artistic conception that musical works are intended to express.

With the continuous development of technology, human research on music has also deepened. The acoustic characteristics of music can affect people’s emotions and psychological states. Conjugated materials, as an emerging material, provide new ideas and methods for conveying specific emotional sound effects in music performance. Conjugated materials have great potential in conveying specific emotions and sound effects in music performance. By deeply studying the acoustic properties of conjugated materials and exploring their applications in music, new ideas and methods can be brought to music creation and performance, making music more precise in conveying specific emotions and emotions. The innovation of this article lied in: ① Conjugated materials are a class of materials with special structures and properties, whose acoustic properties can be regulated to achieve specific emotional sound effects. This material provides novel expressive capabilities that traditional materials cannot achieve. ② The experimental comparison in this article not only compared conjugated materials with one traditional material, but also compared and analyzes the effects of multiple traditional materials, highlighting the outstanding acoustic characteristics of conjugated materials.

## 2 Acoustic properties of conjugated materials

**FIGURE 1:**
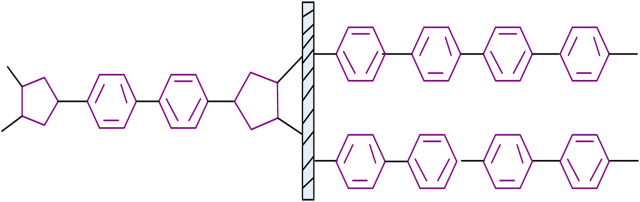
Molecular structure of conjugated materials.

Conjugated materials are materials with special acoustic properties, which are composite materials composed of two different materials (Li et al., 2019). These two materials have opposite dielectric constants and magnetic permeability, so their reflection and transmission characteristics are conjugate to each other, also known as reverse materials. Conjugated materials can counteract some acoustic defects of traditional materials, such as attenuation, reflection, and dispersion, making them widely used in acoustic isolation, absorption, and propagation. The molecular structure of the conjugated material is shown in Figure 1.

The acoustic properties of conjugated materials are achieved by designing their band structure and molecular structure, and molecules with different characteristics can be synthesized through organic chemistry methods (Shao, 2019). These molecules can have different electronic structures and undergo intramolecular electronic transitions within a specific frequency range, resulting in significant acoustic properties.

### 2.1 Acoustic impedance

Acoustic impedance refers to the degree of resistance of materials to sound waves during their propagation, and it is the ratio of sound pressure to sound velocity. Acoustic impedance can affect the propagation and reflection characteristics of sound waves, which is crucial for optimizing acoustic performance. Specifically, the maximization or minimization of acoustic impedance can be achieved by adjusting the thickness and density of the material, while the acoustic impedance of conjugated materials can achieve optimal acoustic performance by adjusting the thickness and density of the material. The acoustic impedance can be calculated by the ratio of sound pressure to sound velocity, as shown in Eq. 1:

$$Z=\frac{P}{v}$$ (1)

Among them, *Z* represents acoustic impedance; *P* represents sound pressure, and *v* represents sound speed; the higher the *Z* value, the greater the resistance to sound propagation.

When sound waves propagate from one medium to another, mismatched acoustic impedances at the interface can cause some of the sound waves to reflect back to the original medium, which is called reflection phenomenon. To minimize reflection, acoustic performance can be optimized by matching the acoustic impedances of the two media.

### 2.2 Sound absorption performance

Conjugated materials also have significant sound absorption properties, as their molecular structure can effectively absorb the energy of sound waves (SuZhengDeng, 2019). Therefore, it has a wide range of applications in the field of acoustics, such as making acoustic lenses, piezoelectric sensors, and acoustic isolators. When sound waves propagate in conjugated materials, electrons in the molecular structure can interact with the energy of sound waves, resulting in resonance absorption. Resonant materials have a better energy absorption effect than ordinary materials, so conjugated materials have a wide range of applications in the field of acoustics (Ruickij et al., 2020). The calculation formula for the sound absorption coefficient is:

η=1−R−T (2)

Among them, *R* represents the reflection coefficient of sound waves, and *T* represents the transmission coefficient of sound waves.

Conjugated materials usually have good sound absorption performance, and their molecular structure and combination method can effectively absorb and dissipate the energy of incident sound waves. Conjugated structures can interact with sound waves at resonant frequencies, converting the energy of sound waves into thermal energy or other forms of energy. This energy conversion process would lead to the attenuation of sound waves, thereby improving the sound absorption coefficient. By adjusting the molecular structure and combination method of conjugated materials, the sound absorption performance can be changed. For example, the conjugation length and functional groups within the molecule of conjugated polymers can be adjusted to improve resonance absorption effect and energy dissipation. At the same time, conjugated materials can also be combined with other sound absorbing materials to form composite materials, further improving sound absorption performance.

### 2.3 Propagation speed

The propagation speed is the speed at which sound waves propagate in a material, and the propagation speed of conjugated materials is usually higher than that of traditional materials, thereby improving acoustic performance. The propagation speed is related to factors such as the density, pressure, and temperature of the medium, so the propagation speed of different materials also varies. Common media include air, water, solids, and conjugated materials. The propagation speed of conjugated materials can be expressed by Eq. 3:

$$S=\frac{c}{\sqrt{\varepsilon }}$$ (3)

Among them, *S* represents the propagation speed of the conjugated material (in meters/second); *c* represents the speed of light in a vacuum; *ε* represents the dielectric constant of the conjugated material.

**FIGURE 2:**
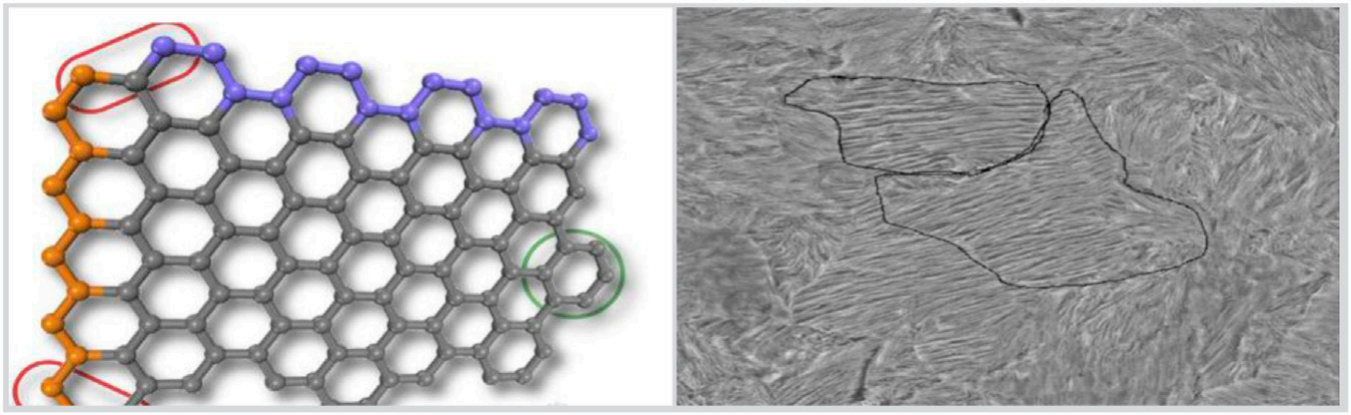
Schematic diagram of conjugated materials with defects.

Conjugated materials are polymer materials with special molecular structures, which have excellent conductivity and acoustic properties. Due to the more uniform arrangement of the molecular structure of conjugated materials, the propagation speed of sound waves in them is usually higher than that of traditional materials, thereby improving acoustic performance. The acoustic performance of conjugated materials is influenced by their molecular structure. Normal polymer materials have some impurities or defects in their molecular structure, which can cause scattering and attenuation of sound waves when propagating within them. The schematic diagram of the conjugated material with defects is shown in Figure 2.

The left side of Figure 2 shows the molecular structure of the conjugated material, with defects present, while the right side of Figure 2 shows the defect conjugated material under an electron microscope.

### 2.4 Performance testing of conjugated materials

#### 2.4.1 Sound absorption performance test

Sound absorption performance testing can be measured using the reflection method, which requires the preparation of an acoustic chamber or acoustic isolation box, as well as a sound source and a receiver (such as a microphone). The test setup needs to be calibrated prior to performing the test. The sample of conjugate material to be tested is fixed in the test position within the acoustic chamber and a standardized acoustic signal is emitted to the conjugate material via a sound source. It is necessary to ensure that the receiver is kept at a distance from the sample surface.

Metal materials, plastics, and wood of the same dimensions were selected for sound absorption tests. The unit of frequency is Hertz (Hz) The experimental parameters are shown in Table 1.

**TABLE 1:** Experimental parameters.

Serial number	Parameter	
1	Thickness	25 mm
2	Frequency range	20 Hz–200 Hz
3	Testing environment	Sound insulation room 1 (poor sound insulation effect)
		Sound insulation room 2 (good sound insulation effect)

In Table 1, the thickness of the metal materials, plastic, and wood selected for the experiment was 25 mm. The experiment was conducted in two soundproof rooms because the differences in material properties under different environments can be compared.

This article used the sound absorption coefficient as an evaluation indicator for sound absorption performance. The sound absorption coefficient is between 0 and 1, and the closer the sound absorption coefficient is to 1, the better the material’s ability to absorb sound waves. Comparing it with traditional metal materials and plastics can evaluate whether conjugated materials have advantages in sound absorption performance.

**FIGURE 3:**
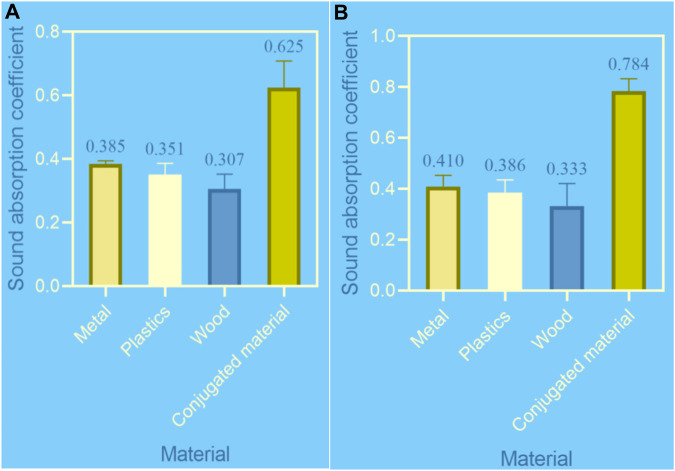
Average sound absorption coefficient of different materials under different sound insulation rooms. **(A)** Average sound absorption coefficient of different materials under sound insulation room 1. **(B)** Average sound absorption coefficient of different materials under sound insulation room 2.

When the distances are 20, 40, 60, 80, and 100 cm, the average sound absorption coefficients of different materials in different sound insulation rooms are shown in Figure 3 (the horizontal axis of Figure 3 represents different materials, namely, metal, plastic, wood, and conjugated materials, while the vertical axis represents the sound absorption coefficient).

In Figure 3A, the average sound absorption coefficients of metal, plastic, wood, and conjugated materials under soundproofing chamber 1 were 0.385, 0.351, 0.307, and 0.625, respectively.

In Figure 3B, the average sound absorption coefficients of metal, plastic, wood, and conjugated materials under soundproofing room 2 were 0.410, 0.386, 0.333, and 0.784, respectively.

Compared to in soundproofing room 1, the average sound absorption coefficient of metal materials under soundproofing room 2 increased by $6.5\%(\frac{0.410-0.385}{0.385}=6.5\%)$ , and the average sound absorption coefficient of conjugated materials increased by $25.4\%(\frac{0.784-0.625}{0.625}=25.4\%)$ .

Whether it was soundproofing room 2 or soundproofing room 1, the average sound absorption coefficient of conjugated materials was higher than that of metals, plastics, and wood.

Wood is the main structural material for many musical instruments, such as woodwind and stringed instruments. Due to its density and sound wave transmission performance, its ability to absorb noise is relatively low. Metal instruments such as brass and percussion instruments are usually made of metal, which has high acoustic reflection and transmission performance, but has poor ability to absorb acoustic energy. Some musical instruments, such as harmonicas and plastic tuning forks, use plastic as the main material. Compared to conjugated materials, ordinary plastic has limited sound absorption performance and is prone to producing echoes and noise.

The material selection of musical instruments is not only based on sound absorption performance, but also on factors such as timbre, resonance characteristics, and appearance (Roche et al., 2021). When manufacturing musical instruments, sound-absorbing materials such as conjugated materials or other sound-absorbing materials can be added at appropriate locations to improve the sound-absorbing performance of the instrument.

#### 2.4.2 Propagation performance testing

**FIGURE 4:**
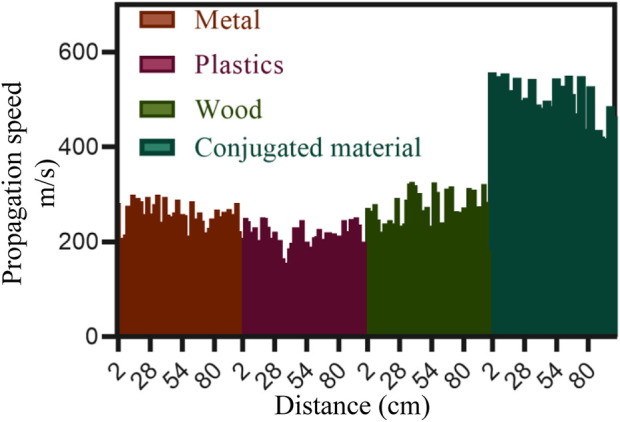
Propagation velocity of different materials at different distances (m/s).

Propagation performance testing can be measured using the sound wave transmission spectrum method. A sound wave generator emitted a constant frequency and amplitude sound wave signal, and the time of sound wave propagation from the transmitter to the receiver was recorded. The propagation time can be measured using a timer or data acquisition device. In order to save time, the experiment was only conducted in soundproof room 2 at a distance of 100 cm. The propagation velocity (m/s) of different materials at different distances is shown in Figure 4 (the abscissa represents the distance in cm, and the ordinate represents the propagation velocity in m/s).

In Figure 4, among metals, plastics, wood, and conjugated materials, the propagation speed of conjugated materials was the fastest, generally above 400 m/s, while the propagation speed of metals, plastics, and wood was all within 400 m/s.

The propagation performance of traditional materials such as metals, plastics, and wood is mainly influenced by the properties of the material itself, while the propagation performance of conjugated materials is more dependent on their special structure and design. Conjugated materials can achieve precise control of sound waves by adjusting their structure and composition, such as reflection, transmission, absorption, etc., with greater flexibility and adjustability. In terms of propagation performance, conjugated materials have more advantages compared to traditional materials, which can achieve more accurate, efficient, and controllable sound wave propagation, and have broad application potential.

## 3 Exploration of acoustic properties of conjugated materials in music performance

### 3.1 Resonance frequency adjustment

By selecting different conjugated materials and their thickness and structure, resonance frequencies can be adjusted to match specific emotions. Resonance frequency refers to the resonance frequency generated by a resonant cavity when subjected to external excitation, which is one of the inherent characteristics of a resonant body. The resonant frequency is related to the length and sound velocity of the effective resonant cavity. A larger acoustic impedance and higher sound velocity can increase the resonant frequency, while better sound absorption performance can reduce the resonant frequency. The resonance frequency is:

$$f=\frac{v}{2L}$$ (4)

Among them, *L* represents the length of the effective resonant cavity, and *v* represents the speed of sound.

Conjugated materials with louder impedance and higher sound velocity can increase resonance frequency, producing bright and light sounds. Conjugated materials with good sound absorption properties can reduce resonance frequency and produce deep and majestic sounds.

Thicker conjugated materials can generate lower resonance frequencies, which are suitable for expressing deep and passionate emotions; thin conjugated materials can generate higher resonance frequencies, making them suitable for expressing bright and cheerful emotions. The resonant frequency is closely related to the size and shape of the resonant cavity. By adjusting the thickness of the conjugated material, the size of the resonant cavity can be changed, thereby adjusting the resonant frequency. The length formula of the resonant cavity is:

$$L=\frac{n\lambda }{4}$$ (5)

Among them, *n* represents the number of wave nodes in the resonant cavity, and *λ* represents the wavelength.

The acoustic characteristics of conjugated materials play a crucial role in adjusting the resonant frequency. Higher impedance and sound velocity can increase the resonant frequency, and the thickness and structural design of conjugated materials are also important factors in adjusting the resonant frequency. By comprehensively considering factors, a good match between music emotions and resonance frequency can be achieved, enhancing the emotional expression and infectivity of music (ZhangMengZhang, 2022).

### 3.2 Sound intensity adjustment

**FIGURE 5:**
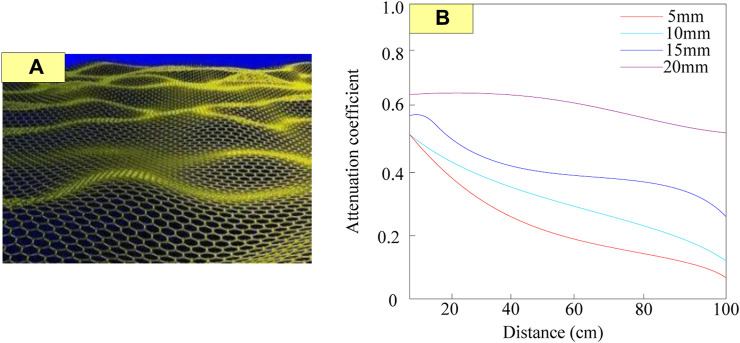
Attenuation effect of conjugated materials. **(A)** Conjugated materials that absorb sound waves. **(B)** Attenuation effect of conjugated materials at different distances and densities.

By adjusting parameters such as thickness and density of conjugated materials, sound attenuation or enhancement can be achieved. Adjusting the thickness of conjugated materials can have a significant impact on the propagation of sound. When sound waves encounter conjugated materials, a portion of the sound energy would be absorbed, scattered, or reflected. The closer the attenuation coefficient is to 1, the better the attenuation effect. Thicker conjugated materials can provide longer paths and increase the propagation time of sound waves in the material, thereby enhancing the attenuation effect of sound, as shown in Figure 5 (in Figure 5B, the abscissa represents distance in cm, and the ordinate represents attenuation coefficient).

Figure 5A shows the conjugated material that absorbs sound waves, while Figure 5B shows the attenuation effect of conjugated materials on sound at different distances and densities. It can be observed that the thicker the conjugated material, the better the effect.

Adjusting the density of conjugated materials can also have an impact on the propagation of sound. A higher density means that there is more mass and energy inside the conjugated material to absorb the vibration of sound waves, thereby increasing the attenuation effect of sound.

The attenuation of sound energy is related to the attenuation coefficient of conjugated materials and the propagation distance of sound waves. By adjusting the attenuation coefficient of conjugated materials, the attenuation of sound can be increased, gradually weakening the sound during propagation. A higher attenuation coefficient would lead to significant loss of sound energy, thereby achieving the attenuation effect of sound. At the same time, the propagation distance of sound waves would also affect the degree of sound attenuation. The longer the propagation distance, the more obvious the attenuation effect. The attenuation of sound energy is related to the propagation distance of sound waves and the attenuation coefficient of conjugated materials:

$$A=10\cdot \log\frac{1}{1+\alpha d}$$ (6)

*A* represents sound energy attenuation; *d* represents the propagation distance of sound waves; *α* represents the attenuation coefficient of the conjugated material.

In addition to thickness and density, other parameters of conjugated materials can also be adjusted to achieve sound attenuation or enhancement, and optimizing parameters can achieve the goal of controlling sound. For example, adjusting the porosity, texture structure, or surface shape of conjugated materials may have an impact on the propagation and absorption of sound.

The density and sound velocity of conjugated materials can affect the magnitude of the attenuation coefficient. A higher density and lower sound velocity of conjugated materials can lead to a higher attenuation coefficient, thereby achieving a greater attenuation effect of sound. At the same time, the sound absorption coefficient also affects the attenuation effect. A higher sound absorption coefficient indicates that the conjugated material has a strong ability to absorb sound waves, which can enhance the attenuation effect. The attenuation coefficient formula of conjugated materials is:

$$\alpha =\frac{\mu }{\rho v}$$ (7)

*ρ* represents the density of the conjugated material, and *μ* represents the sound absorption coefficient of the conjugated material.

In practical applications, different types of conjugated materials can be selected as needed to achieve specific acoustic control effects.

### 3.3 Timbre control

Conjugated materials can change the harmonic distribution and spectral characteristics of sound, thereby adjusting the sensation and texture of the timbre. By selecting different conjugated materials and adjusting their structural parameters, subtle changes in timbre can be achieved to express different emotions (LustigTan, 2020).

The harmonic frequency is related to the harmonic distribution of conjugated materials, and selecting different conjugated materials can change the intensity of the harmonic distribution and the proportional relationship between the harmonic frequency. Some conjugated materials can enhance specific harmonic frequencies, making them more prominent, thereby altering the feel and texture of the timbre. Conjugated materials can absorb specific frequency ranges of sound waves and reduce or eliminate reflections at these frequencies. By selecting different types of conjugated materials, certain frequencies of sound waves can be selectively enhanced or weakened, thereby changing the harmonic distribution of sound. The harmonic frequency formula is:

$$u=\delta \cdot u_{0}$$ (8)

Among them, *u* represents harmonic frequency; *u*_0_ represents the fundamental frequency; *δ* represents the number of harmonics.

The porosity, texture structure, and surface shape of conjugated materials can all affect the scattering and absorption behavior of sound waves. By adjusting these structural parameters, the response of conjugated materials to different frequencies of sound waves can be adjusted, thereby changing the spectral characteristics of sound. For example, in music production, by using different types and thicknesses of conjugated materials on the walls of the recording studio, the reflection behavior of the playing sound can be changed, thereby adjusting the reverberation effect and timbre sensation.

## 4 Application of conjugated materials in music performance

### 4.1 Coating materials

Conjugated materials can be used to coat musical instruments or acoustic equipment to increase their acoustic performance. By altering the harmonics and distribution of the instruments or acoustic equipment through the coating material, it can produce a more harmonious or stimulating timbre (GohThompsonSzeman et al., 2021). This is very helpful for playing music with different styles and expressing different emotions.

It is necessary to select appropriate conjugated coating materials based on the specific characteristics and timbre requirements of the instrument. For each instrument, different conjugated materials can produce different timbre effects. Therefore, when selecting, it is necessary to consider the characteristics of the materials, resonance characteristics, and the overall acoustic design of the instrument. At the same time, it is also necessary to pay attention to the durability and stability of the material to ensure that the wrapping effect is long-lasting and does not affect the performance of the instrument. The coating of conjugated materials can play an important role in musical instruments and acoustic equipment, improving their acoustic performance and changing the timbre. By adjusting the parameters and characteristics of the conjugated material coating, precise control of harmonic distribution and acoustic behavior can be achieved. This can create a more harmonious or stimulating timbre for instruments or acoustic equipment, enhancing the expressive and artistic appeal of performance (Valin, 2020).

It should be pointed out that in the process of selecting and applying conjugated materials, it is necessary to consider the compatibility with musical instruments or acoustic equipment, as well as ensure the best sound quality and playing experience. Therefore, the research and application of conjugated material coating can help promote the development of music and acoustics, and enhance the expression and appreciation of music art.

### 4.2 Outer frame materials

The material of the outer frame has a significant impact on the sound effect of the speaker, and selecting the appropriate material can greatly improve the sound quality of the speaker. A speaker is usually composed of a box and a speaker unit, and the box is the outer shell of the speaker, which directly affects the sound effect of the speaker (FischerSodenThoret et al., 2021). In traditional speaker design, the box is mostly made of wood, but wood can have a certain degree of sound leakage, which affects the sound effect of the speaker. The structure of conjugated materials can effectively suppress sound leakage and improve the sound effect of the speaker.

**FIGURE 6:**
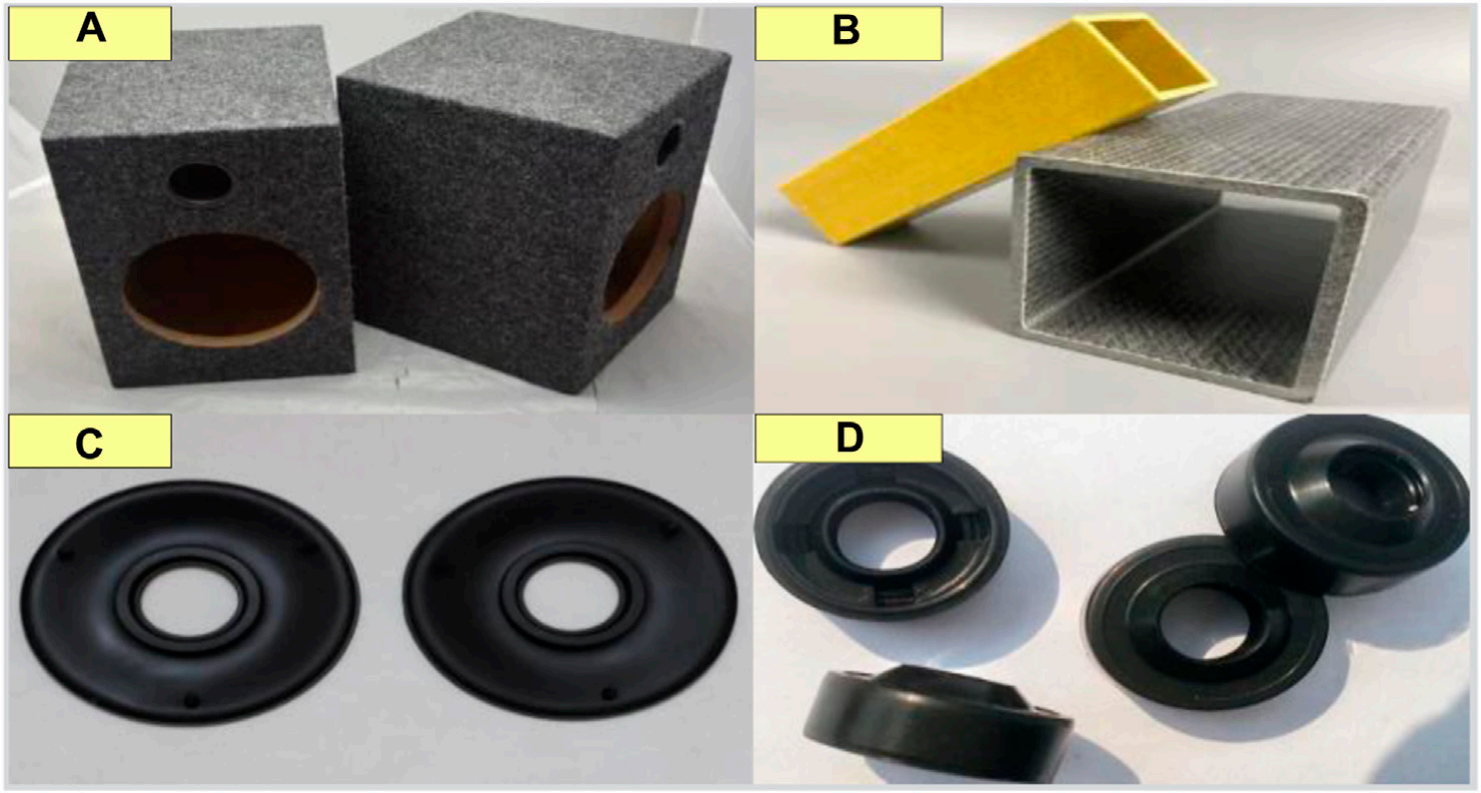
Application of conjugated materials in the speaker frame. **(A)** Fiber reinforced plastics. **(B)** Carbon fiber reinforced plastic. **(C)** Rubber. **(D)** Silicone.

The structure of conjugated materials can be designed as multiple layers or porous structures to increase sound reflection and absorption. In this way, conjugated materials can reduce the leakage of sound from the edges or pores of the box, improving the sound effect of the speaker. Conjugated materials have high sound absorption ability and can absorb sound energy that propagates inside the box. In this way, conjugated materials can reduce the reflection and resonance of sound inside the box, and avoid sound echoes and distortion, thereby improving the sound clarity and accuracy of the speaker, as shown in Figure 6.

In the production of speaker frames, commonly used conjugated materials include fiber reinforced plastic (Figure 6A), carbon fiber reinforced plastic (Figure 6B), and so on. These conjugated materials have excellent strength and stiffness, which can effectively suppress sound leakage and improve the sound effect of the speaker.

Sealing material is a conjugated material with good sealing performance, which can effectively prevent sound leakage. In the production of speaker frames, commonly used sealing materials include rubber (Figure 6C), silicone (Figure 6D), etc. These conjugated materials can fill the gaps and holes in the outer frame structure, forming a closed space and preventing sound from leaking out from the outer frame.

### 4.3 Vibrator materials

Conjugated materials can be used to make vibrators, improving their sensitivity and response speed. Vibrators play a very important role in music performance, directly affecting the quality and effect of music. Using conjugated materials to make vibrators can make them more sensitive and accurate in response to music performance (Friedman, 2018; Li, 2021).

**FIGURE 7:**
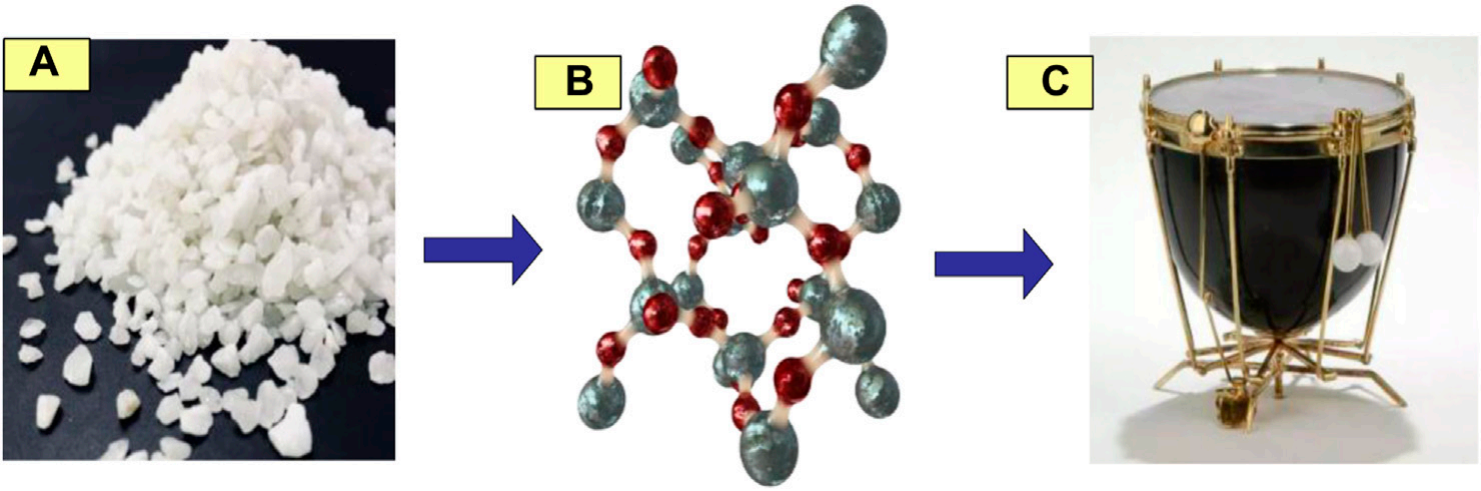
Schematic diagram of quartz material. **(A)** Quartz material. **(B)** Molecular structure of quartz material. **(C)** Instruments made of quartz.

Quartz material has excellent acoustic performance and stable physical properties, making it suitable for making high-frequency vibrators. It has characteristics such as high hardness, high temperature resistance, and bass distortion, and can produce delicate and accurate sound, as shown in Figure 7.

In Figure 7, a is the quartz material, and b is the molecular structure of the quartz material; c is a musical instrument made of quartz material, with a diaphragm on the drum surface.

The diaphragm of a vibrator is the most critical part, directly responsible for converting electrical signals into sound signals. Conjugated materials can be used as materials for the diaphragm, improving its sensitivity and response speed. It has high strength and stiffness, and can quickly respond to audio signals, producing accurate and clear sound. By using a multi layer structure of conjugated materials, the rigidity of the vibrator can be increased, and energy loss can be reduced, thereby improving the efficiency of vibration. The porous structure can increase the surface area of the vibrator and improve the radiation effect of sound.

Through the above methods, conjugated materials can effectively improve the sensitivity and response speed of the vibrator, resulting in a more accurate and clear sound effect. This application can enable the vibrator to better convert electrical signals into sound signals, improving the quality of music.

## 5 Comparison of different material effects experiments

This article selected four representative materials, followed by music works with different emotions, including 28 pieces of happiness, sadness, anger, and calmness, with seven pieces of music for each emotion type.

In the experiment, the music works were played through the speaker, while the conjugated material was placed in front of the speaker. In order to control the experimental conditions, the consistency of factors such as volume, playback time, and speaker distance was maintained.

### 5.1 Comparison of sound effects

#### 5.1.1 Timbre of sound

The transmission of musical emotions largely depends on the characteristics of the sound, such as high notes that make the audience feel bright and light, while low notes that make the audience feel gloomy and heavy. The frequency is usually expressed in Hz, and different frequencies of sound can give people different sensory and auditory effects.

The experiment selected two pieces of music each with the same themes of happiness, sadness, anger, and calmness. The frequency of the sound was statistically analyzed using different materials, with a music frequency range of approximately 20 Hz to 20 KHz. The frequencies of different materials under different types of music are shown in Table 2.

**TABLE 2:** Frequency of different materials under different types of music (Hz).

Types	Number	Conjugated material	Metal	Plastics	Wood
Happy	1	411.49	373.12	279.44	272.86
	2	404.59	287.29	287.28	332.31
Sad	1	393.34	307.00	349.12	274.82
	2	398.98	288.13	327.83	268.62
Anger	1	597.44	559.44	526.88	530.97
	2	556.28	521.53	540.20	515.89
Calm	1	385.20	345.00	329.32	211.70
	2	344.69	250.96	342.67	270.52

In Table 2, the frequency of conjugated materials in happy music was above 400 Hz, and in sad music was above 390 Hz; the frequency of metal in happy music was below 400 Hz, and in sad music it was below 350 Hz; the frequency of plastic in happy music was below 300 Hz, and in sad music it was below 350 Hz; the frequency of wood in happy music was below 350 Hz, and in sad music it was below 300 Hz.

It can be found that the frequency of conjugated materials under different types of music was generally higher than that of metals, plastics, and wood under different types of music.

Duration refers to the duration of a sound, which refers to the time it takes from the beginning to the end. Duration has an impact on the fullness and duration of the sound. The longer the duration within a certain range, the better the sound quality and the greater the impact on emotional transmission. The duration of different materials under different types of music is shown in Table 3.

**TABLE 3:** Duration of different materials under different types of music (s).

Types	Number	Conjugated material	Metal	Plastics	Wood
Happy	1	39.33	29.86	28.44	19.64
	2	41.29	26.45	24.06	18.21
Sad	1	39.99	22.50	21.56	15.24
	2	43.22	22.46	25.85	16.76
Anger	1	36.79	23.08	29.56	18.59
	2	37.76	20.15	33.23	22.72
Calm	1	39.08	27.36	35.91	23.49
	2	40.13	21.35	22.79	24.99

In Table 3, the duration of conjugated materials in happy music was over 35 s, while the duration of metals in happy music was below 30 s; the duration of plastic in happy music was less than 30 s, while the duration of wood in happy music was less than 20 s.

For the transmission of musical emotions, the louder impedance of conjugated materials can reduce the reflection of sound waves and improve the propagation effect of sound waves in conjugated materials, making music easier to convey to listeners and enhancing the emotional expression of music. The sound speed of conjugated materials also has a certain impact on the transmission of musical emotions, and sound speed refers to the speed at which sound waves propagate in the medium. Conjugated materials typically have high sound velocities. A higher sound speed means that sound waves propagate faster in conjugated materials, making music sounds clearer and sharper, and able to more accurately convey music emotions to listeners.

The sound absorption performance of conjugated materials also plays an important role in the transmission of music emotions. Conjugated materials usually have good sound absorption performance, which can effectively absorb and dissipate the energy of incident sound waves, reducing echoes and interference. In music performance and recording environments, the use of conjugated materials can reduce noise and reverberation, making the music more pure and clear, thereby enhancing the listener’s perception of music emotions.

#### 5.1.2 Loudness of sound

Conjugated materials can improve the sound effect of a speaker, thereby improving the loudness and clarity of the sound. In music performance, loudness and clarity are very important indicators that directly affect the quality and effectiveness of music. The application of conjugated materials can make speakers more sensitive and accurate in response to music performance, thereby improving the loudness and clarity of sound.

**FIGURE 8:**
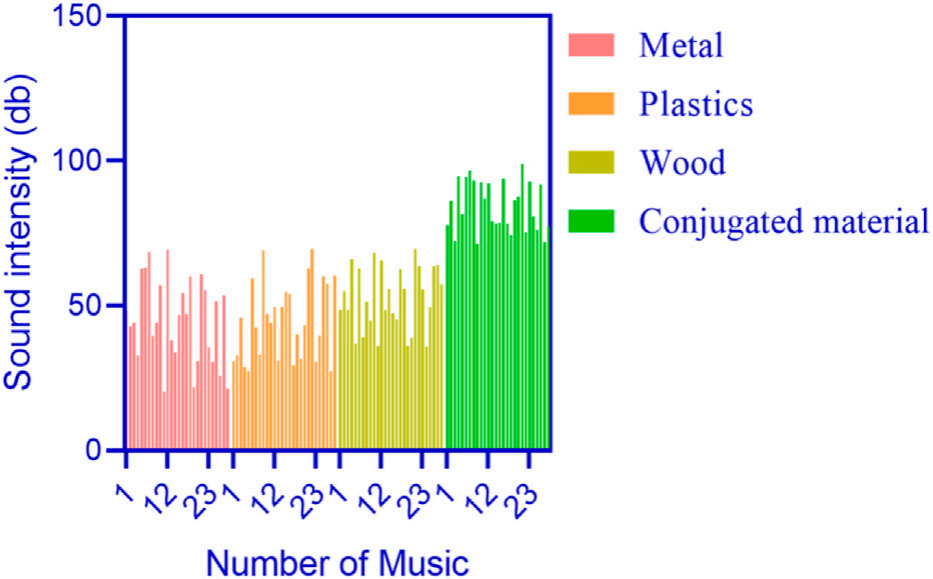
Sound intensity statistics of different materials.

Sound intensity refers to the intensity of a sound, usually expressed in decibels (db), which reflects the intensity or volume of the sound. A higher sound intensity means a greater amount of sound energy, resulting in a higher volume. The sound intensity statistics of 28 pieces of music under different materials were conducted, as shown in Figure 8 (where the horizontal axis represents the number of music and the vertical axis represents the sound intensity in db).

In Figure 8, the overall sound intensity of 28 pieces of music under conjugated materials was above 50 db. Although some of the 28 pieces of music under metal, plastic, and wood had a sound intensity exceeding 50 dB, many were within 50 dB.

Figure 8 shows that the sound intensity of 28 pieces of music under conjugated materials was generally higher than that under metal, plastic, and wood.

#### 5.1.3 Sound stability

**FIGURE 9:**
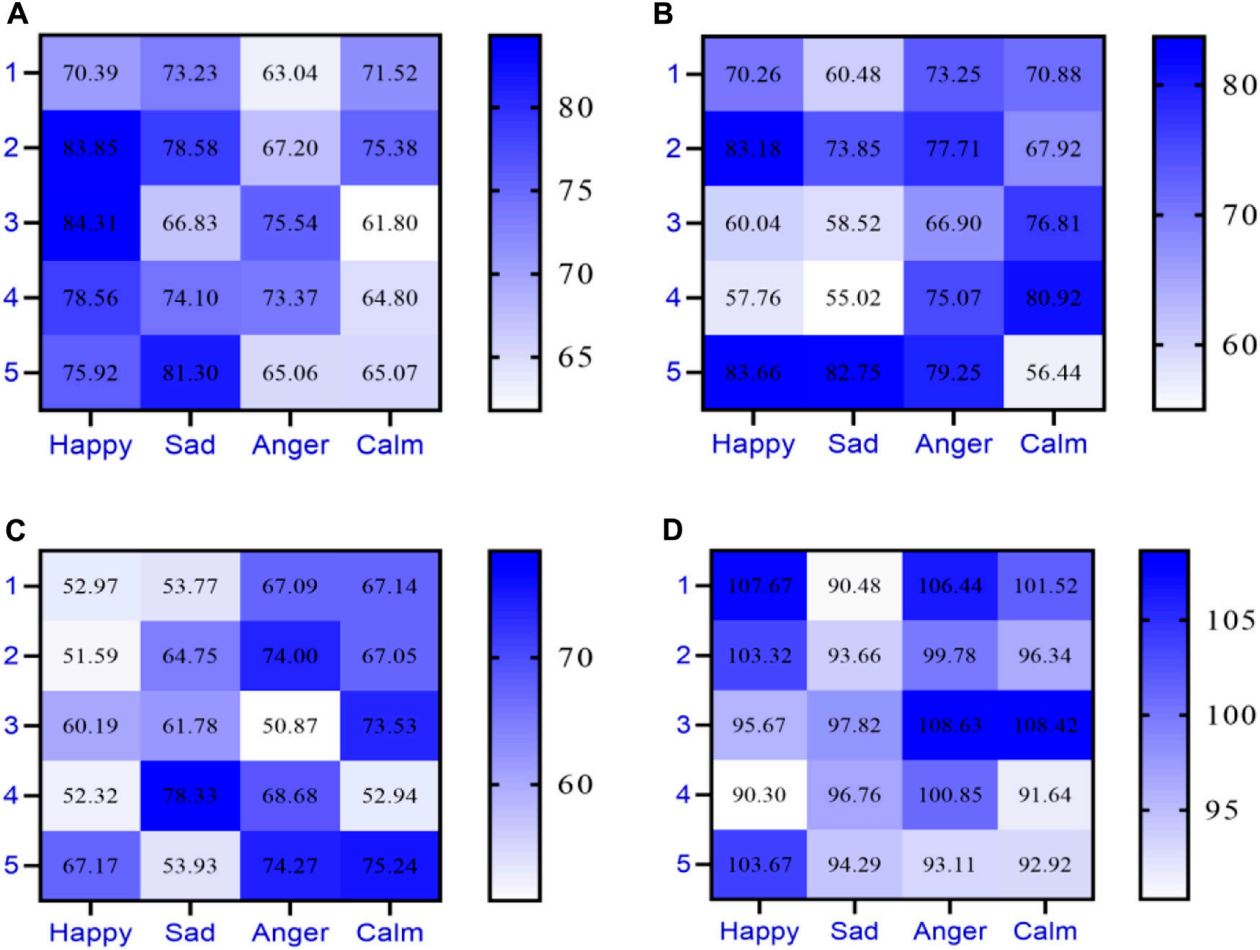
Sensitivity of speaker vibrators made of different materials. **(A)** Sensitivity of metal speaker vibrator. **(B)** Sensitivity of plastic speaker vibrator. **(C)** Sensitivity of wood speaker vibrator. **(D)** Sensitivity of conjugated Material speaker vibrators.

Five pieces of music with different emotions were selected for sound stability testing under vibrators made of different materials. The stability of the sound was tested through the sensitivity of the vibrator. The sensitivity unit of instrument vibrators is usually decibels per watt per meter (db/w/m), with low sensitivity below 70 db/w/m and high sensitivity above 90 db/w/m. The sensitivity of speaker vibrators made of different materials is shown in Figure 9 (the horizontal axis of Figure 9 represents different types of music, namely, happiness, sadness, anger, and calmness, while the vertical axis represents five pieces of music each).

Figure 9A shows the sensitivity of a metal made speaker vibrator, with a maximum of 84.31 db/w/m. Figure 9B shows the sensitivity of a speaker vibrator made of plastic, with a maximum of 83.66 db/w/m. Figure 9C shows the sensitivity of the speaker vibrator made of wood, with a maximum of 78.33 db/w/m. Figure 9D shows the sensitivity of the speaker vibrator made of conjugated materials, with a maximum of 108.63 db/w/m.

Among metals, plastics, wood, and conjugated materials, the sensitivity of speaker vibrators made of conjugated materials was the highest, and the sensitivity of speaker vibrators made of conjugated materials was above 90 db.

### 5.2 Comparison of emotional effects

#### 5.2.1 Emotional transmission

Eight participants were selected, and they were required to rate the emotions conveyed by different types of music (seven pieces of each type) under instruments made of different materials to evaluate their ability to convey different emotions. The average score of the emotional transmission of different materials was calculated, as shown in Table 4.

**TABLE 4:** Average score of emotional transmission for different materials.

Types	Conjugated material	Metal	Plastics	Wood
Happy	87.43	72.74	73.34	71.86
Sad	93.10	67.17	66.23	72.78
Anger	94.51	73.55	71.82	65.28
Calm	90.96	74.73	68.87	65.92

In Table 4, the average scores of participants for happy, sad, angry, and calm music on instruments made of conjugated materials were all above 85 points, while the average scores for happy, sad, angry, and calm music on instruments made of metal, plastic, and wood were all below 80 points.

The average score of happy, sad, angry, and calm music on instruments made of conjugated materials was higher than that on instruments made of metal, plastic, or wood, indicating that the acoustic properties of conjugated materials have a significant impact on the transmission of musical emotions.

The effect of music emotion transmission was better under instruments made of conjugated materials. The louder impedance of the conjugated material reduces the reflection of sound waves, which improves the transmission effect of music, and its higher sound speed makes the music sound clearer and more direct. Good sound absorption performance reduces noise and reverberation, enhancing the emotional sensation of music. By selecting appropriate conjugated materials, the acoustic characteristics of the music performance and recording environment can be improved, thereby enhancing the transmission effect of music emotions. This can enable listeners to better integrate into the music and feel the emotions brought by the music.

#### 5.2.2 Emotional expression

The application of conjugated materials in outer frame materials can improve the sound effect of speakers, making music performance more exciting and moving. Choosing appropriate conjugated materials can produce a more immersive music performance effect, thereby better expressing emotions.

**FIGURE 10:**
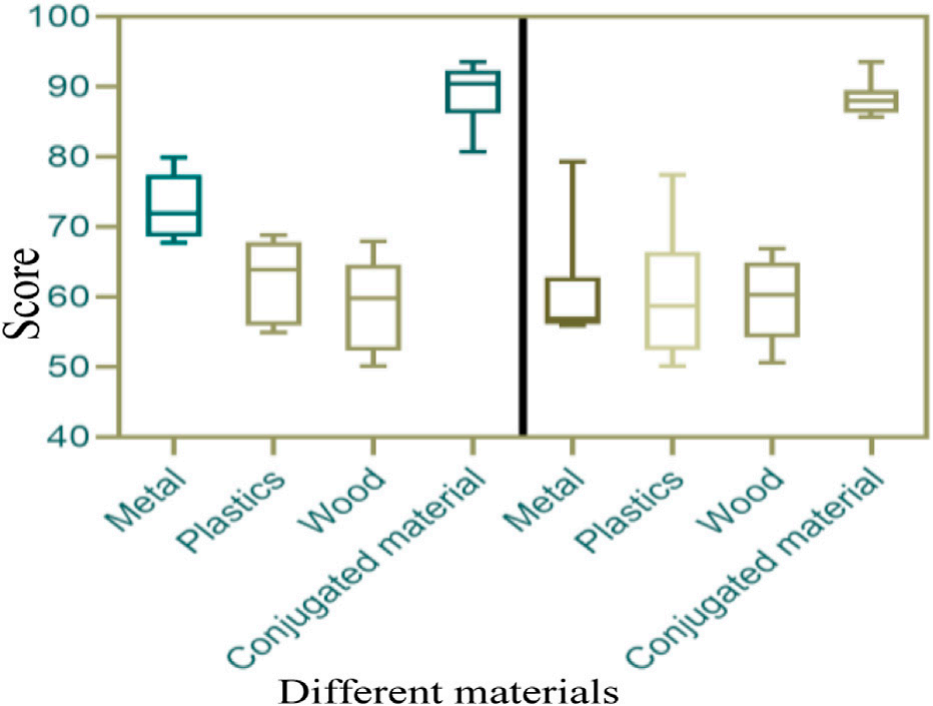
Scores for improving emotional expression under different materials.

Here, only sad and happy music was selected for analysis, and different materials were used to improve emotional expression for score statistics, as shown in Figure 10 (the horizontal axis of Figure 10 represents metal, plastic, wood, and conjugated materials, while the vertical axis represents scores).

In Figure 10, it can be seen that whether it was sad music or happy music, the overall score of emotional expression under conjugated materials (above 80 points) was higher than that under metal, plastic, and wood (overall below 80 points).

The score of emotional expression under conjugated materials was higher than that under metals, plastics, and wood. The acoustic properties of conjugated materials directly affect the sound quality and field effect of speakers, and can enhance the emotional expression effect of music when combined with speakers.

Figure 10 shows that the emotional expression score was higher under conjugated materials. The acoustic properties of conjugated materials directly affect the sound quality and field effect of speakers. Conjugated materials have specific resonance frequencies and resonance peaks, and can enhance sound effects in certain frequency ranges when combined with speaker drivers. By selecting appropriate conjugated materials, the frequency response curve of the speaker can be adjusted to better match the timbre requirements of the music work. For example, for passionate and passionate music works, choosing conjugated materials can enhance the brightness and clarity of high-frequency parts, thereby increasing the energy and impact of the music; For soft and warm music works, conjugated materials can be chosen to increase the richness and warmth of the low-frequency parts, making the music more gentle and graceful.

### 5.3 Discussion

Figure 8 shows that the sound intensity of the music under the conjugated material was higher. Conjugated materials have specific acoustic properties that can effectively guide and disperse sound energy, reducing the occurrence of echoes and stray sounds. For example, using conjugated materials as a substrate and isolation diaphragm inside a speaker can reduce internal reflection and resonance, making the sound more accurate and clear, and avoiding distortion caused by acoustic interference. Conjugated materials can help reduce stray vibrations and resonance phenomena, and improve the stability and rigidity of speaker structures. Applying conjugated materials in appropriate positions can effectively suppress or adjust resonance phenomena and reduce peak occurrences, thereby improving the accuracy and clarity of sound. Conjugated materials can optimize the propagation and reflection of sound waves, improving the acoustic performance of speakers.

Conjugated materials can also improve the sound quality of speakers, making them more balanced and natural. The characteristics of conjugated materials can be adjusted within a specific frequency range, thereby changing the timbre quality and field performance of the speaker. By selecting and applying conjugated materials reasonably, better frequency response balance can be achieved, while maintaining the purity and harmony of the timbre, making music performance more realistic, dynamic, and detailed.

Figure 9 shows that the sensitivity of the speaker vibrator made of conjugated materials was higher. Conjugated materials can improve the sensitivity of vibrators, thereby improving the stability of sound. Stability is a very important indicator in music performance, directly affecting the quality and effectiveness of music. Using conjugated materials to make vibrators can make them more sensitive and accurate in response to music performance, thereby improving the stability of sound.

The sensitivity of a vibrator refers to the sound output level generated by a unit of input energy. By applying conjugated materials around or inside the vibrating components of the vibrator, the quality, rigidity, and damping characteristics of the vibrator can be adjusted, thereby improving vibration efficiency and energy transfer, and enhancing the sensitivity of the vibrator. This means that under the same input conditions, the vibrator would be able to produce a larger sound output, allowing music to be conveyed at a higher volume.

Conjugated materials can also improve the response speed of vibrators, which refers to their ability to drive the diaphragm to vibrate rapidly after receiving electrical signals. The special structure and composition of conjugated materials can effectively reduce the inertia and deflection of the vibrator, and reduce the delay and energy loss of vibration transmission, thereby improving the response speed of the vibrator. This means that the vibrator can more accurately follow the changes in the input signal and make corresponding vibration responses, making the sound more accurate and sensitive. By increasing the sensitivity of the vibrator, conjugated materials can make the speaker produce more stable and accurate sound, which can restore the details and expressiveness of music in music performance, making the music sound more realistic, natural, and realistic.

## 6 Conclusion

Music is an important component of human culture and a broad form of art. It has the ability to express and convey various emotions, and being able to convey specific emotions to the audience is an important goal of music performance. With the continuous progress of technology, people’s requirements for music performance technology are also increasing. Conjugated materials are a special type of material with many special acoustic properties. The application of conjugated materials in music performance can help better convey the sound effects of specific emotions. This article analyzed the acoustic properties of conjugated materials, their impact on music performance, and their effectiveness. The conclusion of this article has important implications for the fields of music performance and emotional regulation. In music creation and performance, selecting conjugated materials reasonably can effectively enhance the emotional expression of music, thereby better conveying the emotions that the author wants to express. In the future, materials used in emotional regulation and psychotherapy can also serve as a new treatment method to help alleviate negative emotions and stress, thereby improving psychological quality and quality of life.

## Data Availability

The original contributions presented in the study are included in the article/Supplementary material, further inquiries can be directed to the corresponding author.
